# Physicochemical Characterization and Immunomodulatory Effect of Melanoidins from Vinegar Residue

**DOI:** 10.3390/foods15152607

**Published:** 2026-07-25

**Authors:** Qidan Chen, Nannan Zhang, Yuxuan Yang, Yue Yu, Changying Wang, Deyang Li, Yixie Chen, Man Zhao, Ting Xia

**Affiliations:** 1 School of Life Sciences, Zhuhai College of Science and Technology, Zhuhai 519041, China; yangyuxuan123@stu.zcst.edu.cn (Y.Y.); changying24@mails.jlu.edu.cn (C.W.); deyang25@mails.jlu.edu.cn (D.L.); 2 Zhuhai Jinhai Institute of Supramolecular Materials, Zhuhai Research Institute, Jilin University, Zhuhai 519041, China; 3 Tianjin Engineering Research Center of Microbial Metabolism and Fermentation Process Control, College of Biotechnology, Tianjin University of Science and Technology, Tianjin 300457, China; zn17864896891@163.com (N.Z.); yuyue20030402@163.com (Y.Y.); 18622241062@163.com (Y.C.); zhaoman1017@163.com (M.Z.)

**Keywords:** melanoidins, vinegar residue, physicochemical characterization, antioxidant capacity, immunomodulatory activity

## Abstract

There is currently a need for high-value applications of vinegar residue, a major byproduct generated during vinegar production, in order to benefit the environment and economic development. Melanoidins exist in vinegar residue as brown and bioactive macromolecules which are produced during the vinegar brewing process. However, the physicochemical characteristics and potential immunomodulatory activity of melanoidins from vinegar residue (VRM) have yet to be clarified. In this study, VRM exhibited structural heterogeneity, with a molecular weight of 9005.42 Da. Carbohydrates were the dominant component, accounting for 57.86 ± 3.36% (*w*/*w*), and xylose was the primary monosaccharide, with molar ratios of 53.15%. In addition, microstructural observations showed clear edges and a relatively smooth surface in VRM samples. Spectral analysis with FTIR showed that VRM has broad-spectrum ultraviolet absorption capacity and contains hydroxyl groups and unsaturated structures. Meanwhile, VRM exhibited higher antioxidant capacity than vinegar melanoidins according to ABTS and FRAP. Furthermore, the effects of VRM on immunomodulation were investigated in macrophages. VRM significantly induced phagocytic capacity and the secretion of NO, TNF-α, and IL-6. VRM at 800 ug/mL effectively exerted an immunomodulatory effect, which was associated with the TLR4-mediated NF-κB pathway. These findings demonstrate that VRM has a polysaccharide skeleton cross-linked with proteins and polyphenols, and possesses antioxidant and immunomodulatory properties. It will provide a novel and functional product for the efficient utilization of vinegar residue.

## 1. Introduction

Vinegar residue, a major solid waste generated during vinegar production, is mainly derived from raw materials such as millet, wheat bran, and sorghum [1]. Vinegar residue can cause significant environmental pollution due to its high acidity, abundant organic matter content, and slow decomposition rate. It has been reported that more than 3 million tons of vinegar residue (VR) are produced in China annually [2]. Since VR is characterized by high moisture and low pH, and is rich in lignocellulose and other organic matter, it can cause serious environmental pollution if not properly treated before discharge. At the scale of production, the main disposal methods for VR are landfill and incineration. These methods not only cause secondary pollution to air and soil but also represent a waste of bioresources [3]. Therefore, there is an urgent need to develop alternative disposal methods for VR. Currently, some residues are used as feedstock, compost-amended media, and cultivation substrates. Song et al [4]. found that 40 g/kg vinegar residue fed to laying hens in the diet decreased digesta pH and increased pepsin activities in the gizzard, and affected the gut microflora. Du et al. [5] reported that vinegar residue successfully reduced the population density of *Fusarium oxysporum* f. sp. *cucumerinum*, and effectively controlled the occurrence of cucumber Fusarium wilt. Additionally, vinegar residue as a cultivation substrate elevated pH, and increased the amounts of total nitrogen, available potassium, and organic matter, as well as the bacteria diversity of soil [6]. Therefore, high-value applications of vinegar residue are of great significance for environmental protection and economic development.

Traditional Chinese cereal vinegars are produced using solid-state fermentation using cereals such as sorghum, wheat bran, rice, and millet as raw materials [7]. During brewing, starch is converted to fermentable sugars by enzymes in the starter (Qu), fermented to alcohol, and then mixed with wheat bran, rice hull, and vinegar starter (Pei) to initiate acetic acid fermentation, during which microorganisms produce acetic acid and various flavor compounds. After leaching, the residual solid, known as vinegar residue, is produces as a predominant organic solid waste of the vinegar industry [8]. It has been reported that vinegar residue primarily consists of cellulose (22.96–34.91%), hemicellulose (16.22–39.8%), lignin (9.2–24.78%) and ash (5.62–13.17%) [2]. It also contains various bioactive compounds such as polyphenols, flavonoids, and melanoidins, which are formed during the vinegar brewing process from either raw materials or microbial fermentation [9]. Melanoidins are generated in the final stage of the Maillard reaction during food thermal processes, and present highly heterogeneous brown macromolecules [10]. Melanoidins are polymers formed through the covalent cross-linking of heterocyclic compounds such as furans, pyrroles, and pyridines into three-dimensional networks. These networks intertwine with carbohydrates, proteins and other organic compounds, conferring structural integrity and physical stability to melanoidins [11,12]. In addition, absorbance at 420 nm serves as an indirect indicator of melanoidin, correlating with the development of colored polymers during the final phase of the Maillard reaction [13].

The structural characteristics of melanoidins are closely connected to their biological activities. Li et al. [14] proved that melanoidins from Zhenjiang aromatic vinegar powder (20 ug/mL) significantly decreased the levels of reactive oxygen species and proinflammatory cytokines including tumor necrosis factor-α (TNF-α), interleukin-6 (IL-6), and IL-1β in alcohol induced macrophages, which exhibited antioxidant and anti-inflammatory activities. Another study reported that vinegar melanoidins (VM) exerted gut microbiota-modulating effects during in vitro fermentation, effectively promoting the proliferation of beneficial bacterial taxa, including the phylum *Firmicutes* and the genus *Bifidobacterium* [15]. Song et al. [16] found that 100 mg/kg black garlic melanoidins significantly restored the phagocytosis ability of macrophages and increased B-lymphocyte proliferation in cyclophosphamide-induced immune deficiency, demonstrating the immunomodulatory effect of melanoidins from black garlic. These studies mainly reported that melanoidins from different foods exhibit various biological activities, such as antioxidant, anti-inflammation, immune and gut-microbiota-modulating effects. However, the physicochemical characteristics of melanoidins from vinegar residue (VRM) are still unclear. Whether it has potential immunomodulatory activity remains to be further explored.

In this study, melanoidins were extracted from vinegar residue. Firstly, molecular weight and chemical composition were detected in VRM samples. Then, the microstructure and spectral characteristics were investigated using scanning electron microscopy (SEM), ultraviolet–visible (UV–vis) spectroscopy, and Fourier-transform infrared spectroscopy (FT-IR). Furthermore, the antioxidant and the immunomodulatory activities of VRM were assessed in vitro. The findings will provide a novel strategy for high-value applications of vinegar residue and its potential use as a functional product.

## 2. Materials and Methods

### 2.1. Materials and Chemicals

Vinegar and vinegar residue were provided by Zilin Vinegar Industry Co., Ltd (Taiyuan, China). Glucose was provided by Fuchen Chemical Reagent Co., Ltd. (Tianjin, China). Dialysis bags with a molecular weight cutoff of 3.5 kDa, Folin–Ciocalteu’s phenol reagent, gallic acid, and 2,2-Diphenyl-1-picrylhydrazyl (DPPH) were obtained from Solaibao Biological Technology Co., Ltd. (Beijing, China). Xylose, glucose, arabinose, mannose, galactose, glucuronic acid, rhamnose, and lipopolysaccharide (LPS) were purchased from Sigma-Aldrich (Shanghai, China), and derived from *Escherichia coli* (O111:B4). 2,2′-Azinobis-(3-ethylbenzthiazoline-6-sulphonate (ABTS)), ferric ion reducing antioxidant power (FRAP), the cell counting kit-8 (CCK-8), and neutral red uptake and nitric oxide (NO) detection kits were purchased from Biyuntian Biotechnology Co., Ltd. (Shanghai, China). ELISA kits including TNF-α and IL-6 were purchased from Yuanju Biotechnology Co., Ltd. (Shanghai, China). Primary antibodies against TLR4, MyD88, IκBα, p-p65, and β-actin were purchased from Santa Cruz, Inc. (Santa Cruz, CA, USA). Primary antibodies against p65, p-IκBα and anti-mouse and anti-rabbit recombinant secondary antibodies (H+L) were purchased from Proteintech Group, Inc. (Wuhan, China). All reagents and chemicals were of analytical standard.

### 2.2. Preparation of VRM and VM

The vinegar and vinegar residue were collected from the same production batch by traditional solid-state fermentation to ensure raw material consistency. Three batches of vinegar and vinegar residue from the same year were used to prepare VRM and VM to decrease the variability of materials from batch to batch. VRM and VM were prepared according to a previous study [15]. Briefly, the vinegar residue was mixed with distilled water at 1:20 (*w*/*v*). The liquid from the vinegar residue and the vinegar were centrifuged at 8000 rpm for 20 min, and then concentrated by rotary evaporator at 55–60 °C. After that, two kinds of concentrated solutions were dialyzed at room temperature for 2 days. The remaining retentate with molecular weight greater than 3.5 kDa was collected and freeze-dried to obtain VM and VRM samples. The extraction of VRM and VM was repeated three times. The yields of VRM and VM were 0.10 ± 0.09 g/100 g and 0.82 ± 0.07 g/100 g, respectively.

### 2.3. Determination of Molecular Weight Distribution

VM and VRM samples were dissolved in deionized water and then detected using high-performance liquid chromatography (HPLC) according to a previous study [17]. An Agilent 1260 system equipped with TSK-gel G4000PWXL (300 × 7.8 mm) and a refractive index detector was used. Sodium nitrate solution (0.1 mol/L) with a flow rate of 0.6 mL/min was used as the mobile phase, and the sample injection volume was 20 uL. The column oven and detector temperatures were 40 °C. The molecular weight of melanoidins was determined using dextran standards for calibration.

### 2.4. SEM Observations

The morphologies of VM and VRM were observed using an SEM (SU3800, Hitachi, Tokyo, Japan) equipped with secondary electron detectors. The samples were uniformly spread on the electron microscope stage and coated with gold powder. The samples were then characterized at 500× and 1000× magnification.

### 2.5. Detection of Chemical Composition

#### 2.5.1. Carbohydrate Content, Protein Content, and Total Phenolics Content

The carbohydrate content in VM and VRM was quantified using the phenol–sulfuric acid method [18]. Briefly, 1.0 mL of sample solution was mixed with 1.0 mL of 5% phenol solution, and then 5.0 mL of concentrated sulfuric acid was added rapidly. The mixture was allowed to stand for 20 min at room temperature for color development. The absorbance was measured at 490 nm using a UV–Vis spectrophotometer. Glucose was used as the standard to establish the calibration curve. The protein contents in VM and VRM were determined according to the Kjeldahl method in National Food Safety Standard GB 5009.5-2016 [19]. Total phenolic content was assessed using the Folin–Ciocalteu method [18]. Briefly, an appropriate aliquot of sample solution was mixed with the Folin–Ciocalteu phenol reagent. After a short incubation period, sodium carbonate solution was added to the mixture and allowed to stand for color development at room temperature. The absorbance was measured at 765 nm using a UV–Vis spectrophotometer (Shimadzu Corporation, Kyoto, Japan). Gallic acid was used as the standard to establish the calibration curve.

#### 2.5.2. Monosaccharide Composition

Briefly, 5 mg of VRM sample was added to a hydrolysis tube, followed by 1.0 mL of 2 mol/L trifluoroacetic acid (TFA). Then, the mixture was placed in a constant-temperature oven at 121 °C for 120 min. The TFA was evaporated to dryness using a nitrogen blower at 50 °C, and washed with 1 mL of HPLC-grade methanol followed by nitrogen blowing 3 times. Subsequently, the residue was redissolved in deionized water, and filtered through a 0.22 μm nylon membrane. Finally, the filtrate was used for monosaccharide composition analysis using an ion chromatograph (ICS 5000+, Thermo Fisher Scientific, Waltham, MA, USA) with the Dionex™ CarboPac™ PA20 (150 × 3.0 mm, 10 μm) liquid chromatographic column. Flow rate was 0.5 mL/min, and injection volume was 5 μL. The solvent systems consisted of system A (dd H2O), system B (0.1 M NaOH), and system C (0.1 M NaOH, 0.2 M NaAc) with a gradient program including volume ratios of solution A, B, C as follows: 95:5:0 at 0 min, 85:5:10 at 26 min, 85:5:10 at 42 min, 60:0:40 at 42.1 min, 60:40:0 at 52 min, 95:5:0 at 52.1 min, and 95:5:0 at 60 min. Measurement data were represented by a molar ratio [15].

### 2.6. VM and VRM Spectrometry Analysis

#### 2.6.1. UV–Vis Detection

The UV–vis spectra of the samples were analyzed using a previous method with slight modifications [20]. Briefly, aqueous solutions of VM and VRM were prepared at a concentration of 0.1 mg/mL. The samples were detected using a UV–vis spectrophotometer (UV 3600 Plus, Shimadzu, Kyoto, Japan) and the spectra were recorded across the wavelength range of 200–800 nm.

#### 2.6.2. FTIR Detection

A 0.3 mg sample was combined with 150 mg of KBr powder and processed into pellets through grinding and compression. The chemical structures of VM and VRM were analyzed using an FT-IR spectrometer (Tensor 27, Bruker, Waltham, Germany). Each sample was scanned three times. Spectral data were collected in the range of 400–4000 cm^−1^ at a resolution of 4 cm^−1^.

### 2.7. Antioxidant Capacity Assays

The antioxidant activities of VM and VRM were evaluated using DPPH, ABTS, and FRAP assays, performed in accordance with previously published protocols [15,21]. Briefly, the sample was mixed with DPPH methanolic solution and incubated in the dark at room temperature for 30 min. The absorbance was measured at 517 nm to determine the scavenging activity against DPPH radicals. In addition, the ABTS radical cation (ABTS•+) was generated by reacting ABTS stock solution with potassium persulfate. After incubation in the dark for 5 min, the working solution was adjusted to an absorbance of 0.70 ± 0.05 at 734 nm. The sample was then mixed with the ABTS•+ solution and the absorbance was measured at 734 nm. For the FRAP assay, the ferric-reducing ability was assessed by measuring the formation of a blue ferrous complex at 700 nm under acidic conditions (pH 3.6).

### 2.8. Cell Culture

RAW264.7 cells were obtained from the Type Culture Collection of the Chinese Academy of Sciences (Shanghai, China) and cultured in DMEM high-glucose medium supplemented with 10% fetal bovine serum (FBS) and 1% penicillin–streptomycin in a humidified incubator at 37 °C with 5% CO_2_.

### 2.9. Cell Viability Assay

RAW264.7 cells were seeded into 96-well plates at a density of 5 × 10^4^ cells/cm^2^ and cultured overnight. After treatment for 24 h according to the experimental groups, 10% CCK-8 reagent (Beyotime Biotechnology, Beijing, China) was added to each well. The plates were incubated for 2 h in the dark, and absorbance was detected at 450 nm using a microplate reader (Jinan Laibao Medical Devices Co., Ltd. Shandong, China).

### 2.10. Pinocytic Activity Assay

Macrophages were seeded in 96-well plates at a density of 1 × 10^5^ cells/cm^2^ and cultured overnight. The cells were then treated with different concentrations of VRM for 24 h. The phagocytic activity was subsequently assessed using a neutral red uptake assay kit (Beyotime Biotechnology, Beijing, China). After removing the culture medium, the cells were gently washed once with PBS. Then, 220 μL of neutral red working solution (freshly prepared by mixing neutral red staining solution with DMEM complete medium at a volume ratio of 1:10) was added to each well, and the plate was incubated for 2 h. After incubation, the supernatant was discarded and the cells were gently washed with pre-warmed PBS to remove unabsorbed dye. Subsequently, 200 μL of cell lysis buffer was added to each well, and the plate was shaken at room temperature for 15 min. The absorbance was measured at 540 nm using a microplate reader.

### 2.11. Determination of NO and Cytokines

Macrophages were seeded in 24-well plates at a density of 1 × 10^5^ cells/cm^2^. After treatment for 24 h according to the experimental groups, the cell culture supernatants were collected. The concentrations of NO, TNF-α, and IL-6 were determined using the corresponding NO detection kit and ELISA kits (Beyotime Biotechnology, Beijing, China), strictly following the manufacturer’s instructions.

### 2.12. Western Blot

Macrophages were seeded in 6-well plates at a density of 5 × 10^6^ cells/cm^2^, and incubated with LPS or VRM (200, 400, and 800 μg/mL) for 24 h. The protein expressions of the samples were detected by Western blot according to a previous method [22]. Collected cells from different groups were treated with lysis buffer (200 μL) containing 1 mM PMSF on ice for 20 min and centrifuged at 12,000 rpm for 10 min. Protein concentration was measured using the BCA assay. The extracted proteins were loaded onto 10% SDS-PAGE and subsequently transferred onto a PVDF membrane. After incubation with primary and secondary antibodies, protein bands were visualized using an Odyssey infrared imaging system (Li-Cor, Licoln, NE, USA) and quantified using ImageJ software (version 1.54 g, NIH, Bethesda, MD, USA).

### 2.13. Statistical Analysis

Data were presented as mean ± standard deviation (S.D), and each set of experimental data was repeated three times. The experimental results and their variations were processed using GraphPad Prism (GraphPad Prism, 8.0, San Diego, CA, USA). The normality of data was evaluated using a Shapiro–Wilk test, and the homogeneity of variance was estimated using Levene’s test. Then, one-way analysis of variance (ANOVA) followed by Tukey’s post hoc test was used for comparisons among three or more groups, and Student’s *t*-test was applied for comparisons between two groups. Different lowercase letters in tables indicated statistically significant differences (*p* < 0.05). *p* values < 0.05 were considered to be statistically significant.

## 3. Results

### 3.1. Analysis of Molecular Weight Distribution of VRM

**Figure 1:**
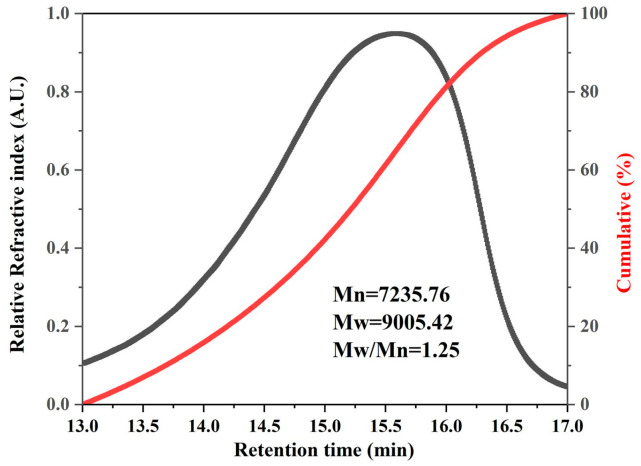
Molecular weight distribution of VRM. Mn: Number-average molecular weight, Mw: Weight-average molecular weight. Black line represents cumulative distribution curve, and red line represents relative refractive index curve.

The molecular weight distribution of VRM was determined using HPLC. As shown in Figure 1, the peak retention time of melanoidins was 15.59 min, and the molecular weight was about 9005.42 Da based on the calibration curve. The polydispersity index (PDI), defined as the ratio of weight-average to number-average molecular weight (Mw/Mn), was used to evaluate the molecular weight distribution characteristics of the samples [23]. A PDI value more than 1 indicated that the melanoidin sample has a broad molecular weight distribution, reflecting its structural heterogeneity [24]. In this study, the PDI value of VRM was 1.25, which was more than 1. Hu et al. [24] investigated the molecular weight distribution of melanoidins extracted from coffee and found a PDI value of 1.3, which was similar to our results. In summary, the data indicate that VRM presents a heterogeneous mixture of compounds with diverse micromolecular weights.

### 3.2. Chemical Composition Analysis of VRM

The basic composition and monosaccharide profile of VRM are presented in Table 1. Carbohydrates were the main component of VRM (57.86 ± 3.36%), followed by proteins (14.97 ± 0.31%) and phenolic compounds (7.78 ± 0.09%). These results indicate that the basic structure of VRM is formed with carbohydrates as the backbone. It has been reported that melanoidins in fermented grain foods primarily consist of carbohydrates as their main component [21], which was consistent with our result. Research studies have reported that the carbohydrate content in melanoidins from traditional grain-based fermented vinegar ranges from 50% to 80% [15,21,25]. In our study, the carbohydrate content was 57.86 ± 3.36% (*w*/*w*), which was consistent with these findings. In addition, the monosaccharide composition of VRM was further analyzed. The result showed that xylose, glucose and arabinose were the main three monosaccharides in VRM, with molar ratios of 53.15%, 21.33% and 12.81%, respectively. The data implied that xylose was the most abundant monosaccharide in VRM. Grain vinegar residue is a byproduct generated during the fermentation process of vinegar production when using sorghum, wheat bran and rice as raw materials. Vinegar residue contains abundant lignocellulose, and serves as a potential source of monosaccharides such as xylose and glucose [26]. This might contribute to the predominantly carbohydrate content of VRM. Recent studies have analyzed the monosaccharide composition of melanoidins from grain vinegar and found that glucose is the most abundant carbohydrate [15]. In our previous study, glucose accounted for 31.16% in the monosaccharide composition, and was the most abundant carbohydrate in VM [15]. It has been reported that high levels of glucose are present during the vinegar fermentation process, and can provide enough reaction substrates and simultaneously form melanoidins [15]. Meanwhile, xylose is not readily utilized by microorganisms during fermentation [27]. This could explain these differences in monosaccharides between VRM and VM.

### 3.3. Micromorphology Characteristics of VRM

**Figure 2:**
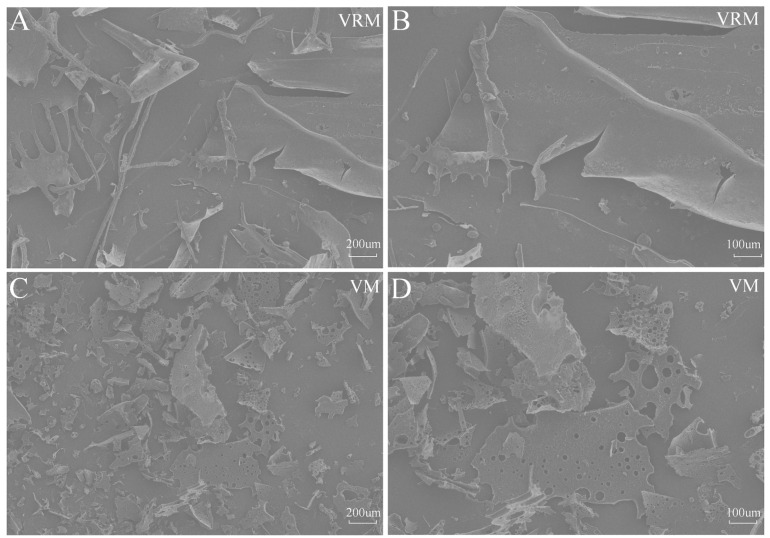
SEM images of VRM (**A**,**B**) and VM (**C**,**D**) with different magnifications.

SEM, as a high-resolution characterization technique, can effectively analyze the microscopic structural features of molecular surfaces [28]. The microstructural features of VRM and VM are shown in Figure 2. Major pieces of VRM samples demonstrated flaking and a compact structure surrounded by some stripe-shaped pieces (Figure 2A). With increased magnification, VRM samples exhibited clear edges and relatively smooth surfaces (Figure 2B). In contrast, VM samples displayed block-shaped and loose structures with different sizes, surrounded by irregular tiny fragments (Figure 2C). With increased magnification, various voids were observed on the rough surface of the blocks in VM samples (Figure 2D). Gryczka et al. [29] reported that lignocellulosic material showed a flat and smooth surface under SEM, which was similar to our results. Lignocellulose primarily consists of cellulose, hemicellulose, and lignin, with xylose and glucose present in the cellulose and hemicellulose fractions [30]. Moreover, xylose cannot be utilized by microorganisms as sufficiently as glucose [31]. Xylose was the most abundant carbohydrate in VRM, which made it challenging for microorganisms to utilize it. This would explain the smooth surface of the compact structure in VRM samples. In contrast, glucose is the most abundant carbohydrate in VM [15], and glucose is consumed by microorganisms as a carbon source during the fermentation process [32]. This may be the reason for the formation of holes on the VM surface of the loose structure through the action of microorganisms during fermentation. Collectively, the change in chemical compositions in vinegar and vinegar residue through fermentation leads to differences in surface morphology between VRM and VM.

### 3.4. Spectral Characteristics of VRM

#### 3.4.1. UV–Vis Spectroscopy Analysis of VRM

**Figure 3:**
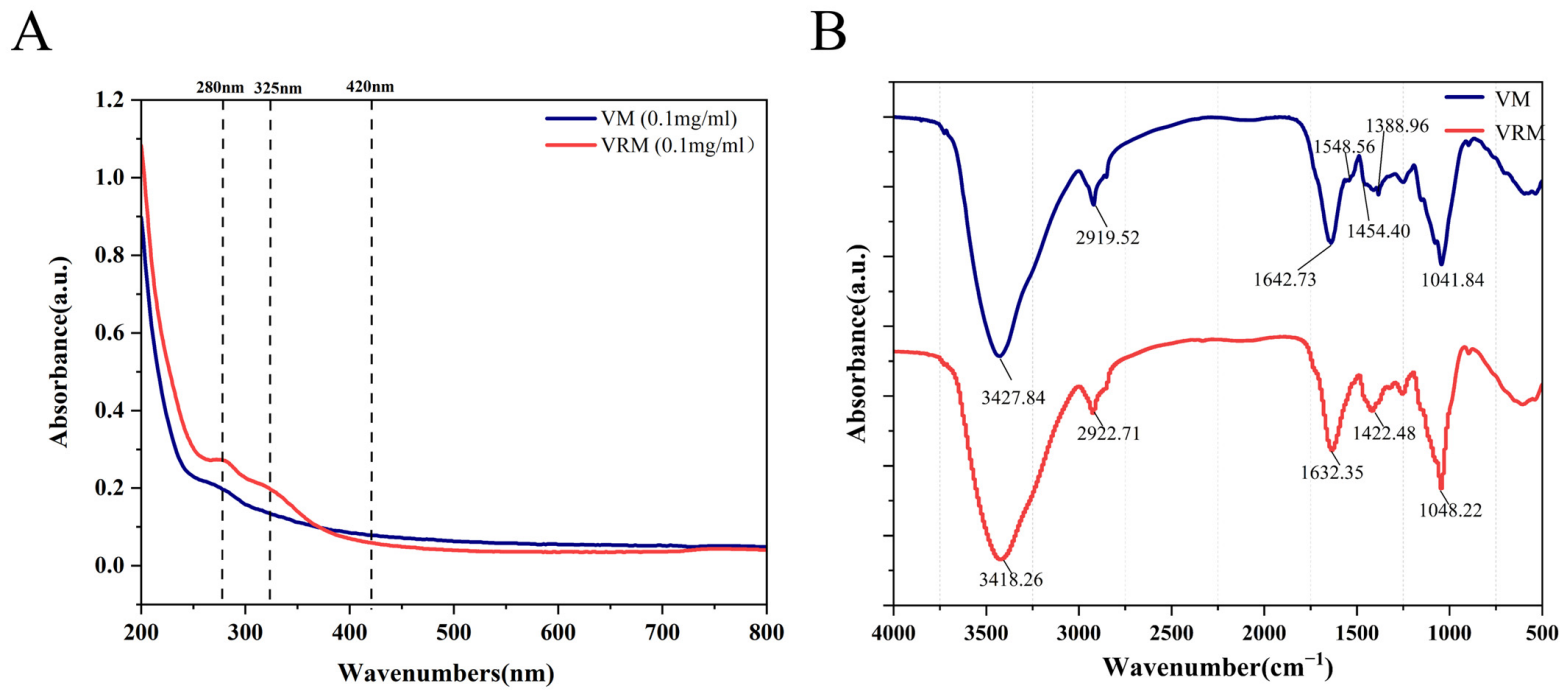
Spectral characteristics of VRM and VM. (**A**) UV-vis absorption spectra, and (**B**) the FTIR spectra.

The UV–vis absorption spectroscopy of VRM and VM samples is shown in Figure 3A. VRM and VM had similar spectrum characteristics between 200 and 350 nm. It has been demonstrated that UV–vis absorption at 200–350 nm is associated with unsaturated aldehydes and ketones or conjugate systems [33]. This suggests that there was an unsaturated structure in VRM and VM samples which contributed to antioxidant capacity due to the electronic transition in the C=C, C=O and N=O bonds. In addition, VRM showed a higher absorption value at 280 nm and 325 nm. It has been reported that the absorption peak at 280 nm is related to proteins and heterocyclic chemicals like furan or pyrrole or conjugated substructures of Maillard reaction (MR)-derived colorants [34]. Additionally, the peak at 325 nm refers to the presence of the benzene conjugates such as flavonoids and chlorogenic acids [33]. Meanwhile, as bioactive compounds, polyphenolics play an important role in antioxidant activities and immune regulation [35,36]. These results indicate that VRM had higher protein and polyphenolic content, which is consistent with the chemical composition in the above data. Moreover, the absorption value of VRM was close to that of VM at 420 nm, which is considered a unique absorption signature of melanoidins [37]. These findings suggest that VRM has similar melanoidin content to VM. VRM is cross-linked with proteins and polyphenols, which have UV absorption capacity and potential antioxidant and immunomodulatory activities.

#### 3.4.2. FTIR Analysis of VRM

In our previous study, the spectral characteristics of VM were investigated using FTIR [15]. We further compared the FTIR spectra of VRM with that of VM in this study. As shown in Figure 3B, VRM and VM both exhibited a broad and intense absorption peak around 3420 cm^−1^. This is associated with the -OH stretching vibration [34], indicating the presence of hydroxyl groups in the melanoidins [38]. Additionally, a small and sharp absorption peak was observed around 2920 cm^−1^ in VRM and VM, related to the stretching vibrations of C-H, -CH_2_, and -CH_3_ groups in aromatic rings, indicating the existence of aromatic compounds in melanoidin samples [39]. The sharp absorption peak around 1640 cm^−1^ refers to the C=O and C=C stretching vibrations, indicating the presence of carboxyl groups [40]. Furthermore, the intense and sharp absorption peak around 1045 cm^−1^ was due to the C-O stretching vibration, representing the existence of glycosidic bonds and ester groups [39]. In the region from 1200 to 900 cm^−1^, some characteristic vibrations were observed in VRM and VM. This could be caused by the presence of stretching vibrations of C-O, C-C and C-N single bonds, bending vibrations of C-H bond, and vibrations caused by phenyl rings [41]. All these indicate the presence of polysaccharide structures in melanoidins. An adsorption band near 1500–1200 cm^−1^ is attributed to the C-N stretching vibration and N-H bending vibration of amide III, indicating the presence of proteins [42]. In this region, the absorption band of VRM showed greater absorption value than that of VM, suggesting that VRM contained more proteins than VM. This was consistent with the result of the UV–vis spectroscopy. Collectively, the results above indicate that VRM has structural features including carbohydrates and proteins, and the existence of hydroxyl groups can contribute to antioxidant capacity.

### 3.5. Analysis of the Antioxidant Capacity of VRM

The antioxidant activities of VRM and VM were assessed using three different methods, and are presented in Table 2. The activities of VRM were 0.87 ± 0.03 mM Trolox/g (ABTS), 3.32 ± 0.17 mM Trolox/g (DPPH), and 0.19 ± 0.02 mM Trolox/g (FRAP). The activities of VRM according to ABTS and FRAP assays were significantly higher than those of VM. It has been demonstrated that the bound polyphenols in food melanoidins contribute significantly to their antioxidant capacity [21,43]. In our previous study, the polyphenol content in VM was 5.98 ± 3.48% (*w*/*w*) [15], which was lower than that in VRM. This trend was consistent with the antioxidant activities of VM and VRM detected by ABTS and FRAP assays in the current study. In the above results, UV–vis spectroscopy showed that the absorption values at 325 nm in VRM were higher than those in VM, which is relevant to phenolic compounds such as flavonoids and chlorogenic acids [33]. Wang et al. [44] also found that phenols continuously contributed to the formation of melanoidins as storage time increased in Monascus vinegar, resulting in an enhancement of antioxidant capacity. This could be the reason for the higher antioxidant activities of VRM compared to VM. However, the DPPH radical scavenging activity of VRM was similar to that of VM (3.55 ± 0.14 mM Trolox/g). It is well known that the ABTS and FRAP methods are used to measure antioxidant activity through electron transfer, while the detection principle of the DPPH assay is the integration of electron transfer and hydrogen atom transfer [45]. Therefore, the discrepancy in antioxidant capacities between VRM and VM may arise from the differences in the assay methodologies. The existence of polyphenolics would explain why the FTIR result for VRM displayed a broad and intense absorption peak around 3420 cm^−1^, suggesting the presence of hydroxyl groups. Hydroxyl groups are contained in polyphenolics and are able to strengthen antioxidant capacity. Moreover, VRM exhibited absorption peaks between 1500 cm^−1^ and 1200 cm^−1^, representing amide II (C–N stretching and N–H deformation) and amide III (C=O stretching, C–N stretching, and N–H deformation) [18]. It has been reported that the Maillard reaction changes the structure of melanoidins, and more unsaturated structures (C=O and C=N) were formed in melanoidins during aging [46]. These unsaturated structures enable melanoidins to have stronger antioxidant capacity by transferring electrons or protons [38]. These results imply that the difference in antioxidant capacities between VRM and VM may be the main cause of electron transfer, which can explain the higher antioxidant capacity of VRM compared to that of VM by ABTS and FRAP.

### 3.6. Effects of VRM on Phagocytic Capacity of RAW264.7 Cells

The viability of RAW264.7 cells was detected using a CCK-8 assay to assess non-toxic concentrations of VRM. As depicted in Figure S1, a marked dose-related enhancement in metabolic activity was observed. No significant variations were detected between VRM-treated groups (25–200 ug/mL) and the untreated control (*p* > 0.05), indicating that these concentrations were non-toxic to the cells. The metabolic activity of cells treated with VRM at 400 and 800 μg/mL was significantly higher than that of the control group (*p* < 0.05), with values of 144.25 ± 11.28% and 151.74 ± 18.88%, respectively. LPS, as an endotoxin, was used as a positive control in immunological activity studies [47]. In this study, the metabolic activity saw a significant increase to 158.32 ± 9.94% in the LPS group (1 ug/mL) compared with the control group, which had results similar to VRM at 800 ug/mL. Therefore, the concentrations of VRM at 200, 400, and 800 ug/mL were selected for subsequent immunological experiments.

**Figure 4:**
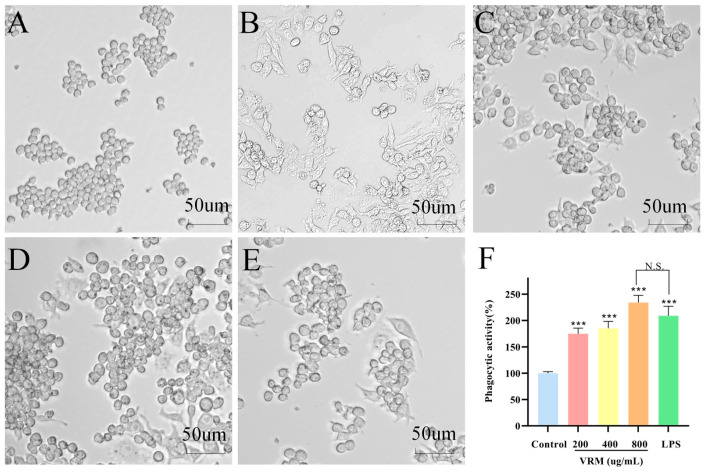
Effect of VRM on cell morphology and phagocytic capacity. (**A**) Control group, (**B**) LPS group, (**C**) 200 ug/mL VRM group, (**D**) 400 ug/mL VRM group, (**E**) 800 ug/mL VRM group, and (**F**) phagocytic activities in different groups. The bar represents 50 um. *** *p* < 0.001 vs. control group. N.S. represents no significant difference.

It is well known that morphological changes in macrophages are closely correlated with their functions, such as activation, polarization, and phagocytic capacity, serving as key phenotypic markers for studying the immunomodulatory activity of macrophages [48]. As shown in Figure 4, a round or slightly flattened oval shape was exhibited in the control group (Figure 4A). After treatment with VRM (200–800 ug/mL), some macrophages displayed a spindle-like and elongated morphology, accompanied by the formation of pseudopodia and an increase in cell size. This morphological change was especially obvious in 800 ug/mL VRM, which was similar to the results for the LPS group (Figure 4B–E). According to the study by Wan et al. [49], the polysaccharide CP80-1 isolated from Cordyceps cicadae was mainly composed of glucose, xylose, and rhamnose. After treatment with CP80-1, RAW264.7 cells showed a spindle-like morphology, enlarged cell volume, and pseudopodia formation. Moreover, these morphological changes became more pronounced as the concentration of CP80-1 increased. This observation was consistent with our results. The morphological alterations can improve contact with and phagocytosis of foreign particles or microorganisms, thereby enhancing the cell migratory and phagocytic capacity [50]. Furthermore, the phagocytic capacity was assessed by the neutral red staining assay. Compared with the control group, all VRM groups (200–800 ug/mL) significantly enhanced phagocytic activity. Notably, the phagocytic capacity was increased to 234.13 ± 13.84% in 800 ug/mL VRM group, which was close to the result for the LPS group. It has been demonstrated that polysaccharides from the gel of Aloe species with molecular weights between 5 and 400 KDa showed the most potent macrophage activity. The reason is that polysaccharides within this range not only possessed high bioavailability but also retained sufficient structural complexity to effectively activate macrophage receptors [51]. In our study, the molecular weight of VRM was about 9 KDa, which fell precisely within this range. These results suggest that VRM, especially 800 ug/mL, obviously activates macrophages and elevates phagocytic activity.

### 3.7. Effect of VRM on NO Release and Cytokine Production in RAW264.7 Cells

**Figure 5:**
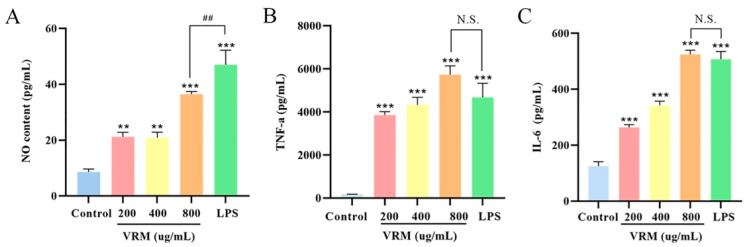
Effect of VRM on NO release and cytokines production. (**A**) NO, (**B**) TNF-α, and (**C**) IL-6. ** *p* < 0.005, *** *p* < 0.001 vs. control group. ^##^ *p* < 0.005 vs. LPS group. N.S. represents no significant difference.

It has been demonstrated that NO is produced by activated macrophages, and suitable NO can modulate macrophage polarization and promote the secretion of cytokines (TNF-α, IL-6). This further regulates immune function and maintains immune homeostasis [52]. As shown in Figure 5A, the NO content was significantly increased in VRM-treated groups, and especially in the 800 ug/mL group. The NO concentration in the 800 ug/mL VRM-treated group was 36.49 ± 0.94 pg/mL, showing a marked reduction compared with the LPS-treated group (47.00 ± 5.20 pg/mL). Geng et al. [53] found that NO production was significantly increased in RAW264.7 cells treated with heteropolysaccharide (SHP) for 24 h. Additionally, the NO content in the SHP group treated with 12.5–100 ug/mL had comparable effects to that in LPS. Lu et al. [54] reported that RAW264.7 cells treated with polysaccharides extracted from black soybean hulls exhibited significantly elevated NO levels at a concentration of 400 ug/mL. NO is one of the key mediators in modulating macrophage immunological activity. The data imply that the increased production of NO after VRM treatment promotes immunomodulation.

In addition, VRM (200–800 ug/mL) significantly elevated TNF-α and IL-6 levels in a concentration-dependent manner compared with the control group. The levels of TNF-α and IL-6 were highest in the 800 ug/mL group, at 5733.92 ± 408.77 pg/mL and 524.83 ± 14.92 pg/mL, respectively. However, TNF-α and IL-6 levels in the LPS group did not significantly differ from those observed in other groups (Figure 5B,C). The secretion of TNF-α and IL-6 was identified as hallmarks of M1-polarized macrophage activation. TNF-α acts as an early secreted proinflammatory cytokine which can enhance macrophage immune functions and induce the expression of antitumor and immunomodulatory mediators [49]. Moreover, IL-6 is strongly implicated in the development of numerous inflammatory and autoimmune conditions [55]. These findings suggest that VRM, especially 800 ug/mL, elevates the secretion of NO and TNF-α and IL-6, which activates macrophages and enhances their immune-regulatory capacity.

### 3.8. VRM-Activated Immune Response Connected with TLR4-Mediated NF-κB Pathway

**Figure 6:**
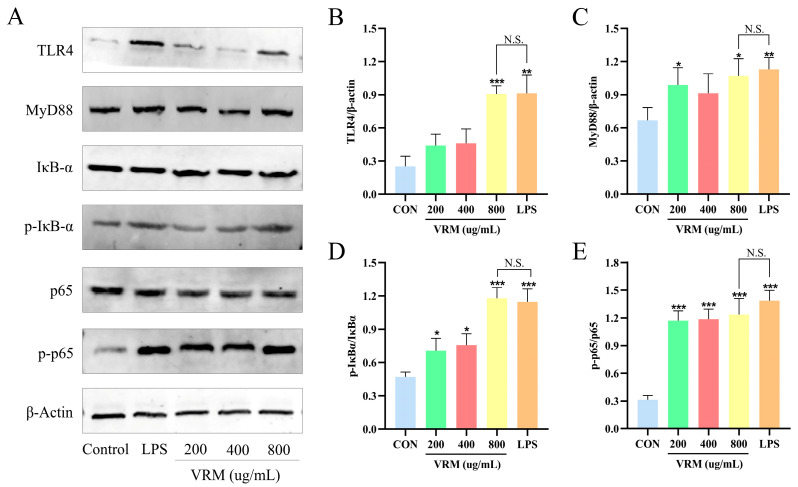
Effect of VRM on TLR4-mediated NF-κB Pathway. (**A**) Protein expression of TLR4/NF-κB pathway, (**B**) the ratio of TLR4/β-actin, (**C**) the ratio of MyD88/β-actin, (**D**) the ratio of p-IκB-α/IκB-α, and (**E**) the ratio of p-p65/p65. * *p* < 0.01, ** *p* < 0.005, *** *p* < 0.001 vs. control group. N.S. represents no significant difference.

To further explore the immunomodulatory mechanism of VRM in RAW264.7 cells, the associated-protein expressions were analyzed by Western blot (Figure 6A). It has been reported that the TLR4-mediated NF-κB signaling pathway is a key regulatory mechanism in immune response [56]. TLR4 is the pattern-recognition Toll-like receptor, which initiates downstream of the NF-κB pathway through adaptor protein MyD88 [57]. When MyD88 is activated, it leads to the phosphorylation and degradation of IκB. Then, p65 is separated from IκB, and translocates into the nucleus to initiates the transcription of target genes which promote iNOS expression, NO production, and the release of cytokines such as TNF-α and IL-6 [58,59,60]. In this study, the expression levels of TLR4 and MyD88 were increased following treatment with VRM at concentrations from 200 to 800 ug/mL, and the expression levels in the 800 ug/mL VRM group were similar to those in the LPS group (Figure 6B,C). Moreover, the ratios of p-IκBα/IκBα and p-p65/p65 were significantly elevated in the VRM group compared with those in the control group. The ratios in the VRM group were close to those in the LPS group (Figure 6D,E). These results indicate that VRM, and especially 800 ug/mL, upregulates the expression of TLR4 and MyD88 and the downstream NF-κB pathway.

Previous studies demonstrated that some polysaccharides from various foods (mushroom, Auricularia polytricha, and Poria Cocos) stimulated macrophages through the corresponding pattern recognition receptor TLR4 on the cell surface instead of by getting into the cell to exert inflammatory and immune regulation [61,62,63,64]. It was found that arabinose, xylose, mannose, and galactose in lentinan have stronger connections with macrophage stimulation activities than other monosaccharides [62]. In our above results, VRM contained abundant xylose and arabinose, which might interact with TLR4 on the cell surface and further activate the NF-κB pathway. Collectively, these results imply that VRM regulates immune response, which is associated with the TLR4-mediated NF-κB pathway.

## 4. Conclusions

**Figure 7:**
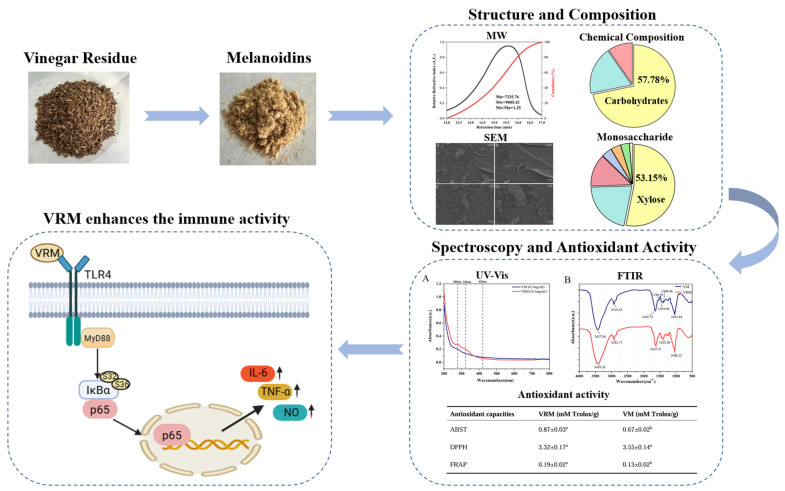
Schematic diagram of physicochemical characteristics and immune modulatory activities of vinegar residue melanoidins.

In summary, the present study revealed the characteristics of VRM and their potential bioactivities (Figure 7). It was found that melanoidins from vinegar residue, as heterogeneous macromolecules, were formed with carbohydrates as the backbone and dynamically bound with proteins and phenolic compounds. Xylose, glucose, and arabinose were the major monosaccharides. The microscopic structure of VRM showed flaking and a compact structure surrounded by some stripe-shaped pieces, and exhibited clear edges and relatively smooth surfaces with an increased magnification. In addition, spectroscopic analysis revealed that VRM had broad-spectrum UV absorption ability and contained carboxyl groups, hydroxyl groups, and proteins in the FTIR spectra. Hydroxyl groups and unsaturated structures in melanoidins contribute to the antioxidant capacity of VRM. This is the first attempt to investigate the effect of VRM on macrophages. It was found that 800 ug/mL VRM significantly upregulated TLR4/MyD88/NF-κB protein expression and induced phagocytic capacity and the production of NO, TNF-α, and IL-6 to promote immune effects. Collectively, these findings indicate that VRM, as a functional macromolecule from vinegar residue, exhibits antioxidant and immunomodulatory activities in vitro. Further research will be conducted to explore the safety, dosage, and immune-regulatory function of VRM in vivo, and to promote high-value applications of vinegar residue in the future.

## Figures and Tables

**Table 1:** Chemical and monosaccharide compositions of VRM.

Chemical Composition (% *w*/*w*)	VRM
Carbohydrates	57.86 ± 3.36
Proteins	14.97 ± 0.31
Phenolics	7.78 ± 0.09
Monosaccharide	composition (mol%)
Xylose	53.15 ± 2.98
Glucose	21.33 ± 0.71
Arabinose	12.81 ± 0.10
Mannose	4.03 ± 0.01
Galactose	3.94 ± 0.10
Glucuronic acid	3.41 ± 0.17
Rhamnose	1.34 ± 0.02

Note: Values are means ± SD (*n* = 3).

**Table 2:** The antioxidant capacities of VRM and VM.

Antioxidant Capacities	VRM (mM Trolox/g)	VM (mM Trolox/g)
ABTS	0.87 ± 0.03 ^a^	0.67 ± 0.02 ^b^
DPPH	3.32 ± 0.17 ^a^	3.55 ± 0.14 ^a^
FRAP	0.19 ± 0.02 ^a^	0.13 ± 0.02 ^b^

Note: Values are means ± SD (*n* = 3), and data with different lowercase letters indicate significant differences (*p* < 0.05) between VRM and VM.

## Data Availability

The original contributions presented in this study are included in the article/Supplementary Material. Further inquiries can be directed to the corresponding authors.

## Supplementary Materials

The following supporting information can be downloaded at: https://www.mdpi.com/article/10.3390/foods15152607/s1, Figure S1: Effect of VRM on the viability of RAW264.7 cells. ** *p* < 0.005, *** *p* < 0.001 vs. control group. N.S. represents no significant difference.

## Abbreviations

The following abbreviations are used in this manuscript:

VRM	melanoidins from vinegar residue
VM	vinegar melanoidins
SEM	scanning electron microscopy
FT-IR	fourier-transform infrared spectroscopy
DPPH	2,2-Diphenyl-1-picrylhydrazyl
ABTS	2,2′-Azinobis-(3-ethylbenzthiazoline-6-sulphonate)
FRAP	ferric ion reducing antioxidant power
LPS	lipopolysaccharide
CCK-8	cell counting kit-8
NO	nitric oxide
TNF-α	tumor necrosis factor-α
IL-6	interleukin-6
PDI	polydispersity index
