# Supplementary material for: Physicochemical Characterization and Immunomodulatory Effect of Melanoidins from Vinegar Residue
Source: Foods. 2026 Jul 25;15(15):2607. doi: 10.3390/foods15152607 (PMC13465938; doi:10.3390/foods15152607)
Supplement: Supplementary file 1 [file foods-15-02607-s001.zip › foods-4412648-supplementary.pdf]

Supplementary material

The physicochemical characterization and immunomodulatory effects of melanoidins from vinegar residue

Qidan Chen [1,2,*], Nannan Zhang [3], Yuxuan Yang [1], Yue Yu [3], Changying Wang [1,2], Deyang Li [1,2], Yixie Chen [3], Man Zhao [3] and Ting Xia [3,*]

[1] School of Life Sciences, Zhuhai College of Science and Technology, Zhuhai 519041, China; yangyuxuan123@stu.zcst.edu.cn (Y.Y.); changying24@mails.jlu.edu.cn (C.W.); deyang25@mails.jlu.edu.cn (D.L.)

[2] Zhuhai Jinhai Institute of Supramolecular Materials, Zhuhai Research Institute, Jilin University, Zhuhai 519041, China

[3] Tianjin Engineering Research Center of Microbial Metabolism and Fermentation Process Control, College of Biotechnology, Tianjin University of Science and Technology, Tianjin 300457, China; zn17864896891@163.com (N.Z.); yuyue20030402@163.com (Y.Y.); 18622241062@163.com (Y.C.); zhaoman1017@163.com (M.Z.)

* Correspondence: qidanchen@jlu.edu.cn (Q.C.); xiating@tust.edu.cn (T.X.)

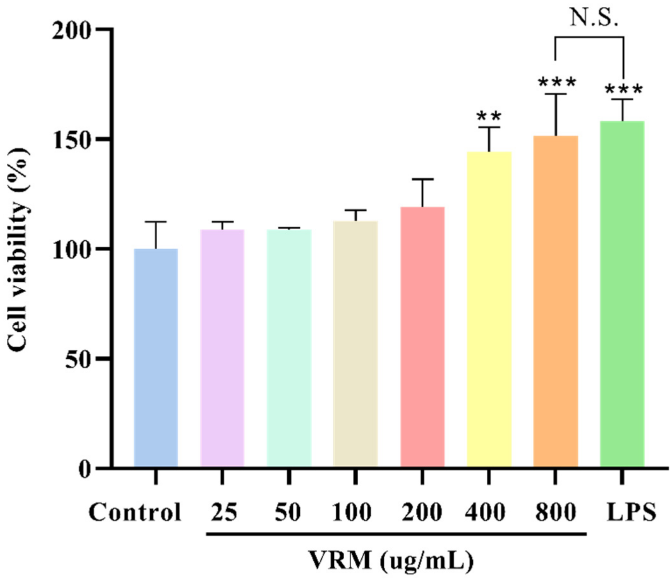

Figure S1. Effect of VRM on the viability of RAW264.7 cells. ** $p < 0.005$, *** $p < 0.001$ vs. control group. N.S. represents no significant difference.
